# Supplementary material for: Transcriptome Analysis Revealed Potential Genes of Skeletal Muscle Thermogenesis in Mashen Pigs and Large White Pigs under Cold Stress
Source: Int J Mol Sci. 2023 Oct 24;24(21):15534. doi: 10.3390/ijms242115534 (PMC10650474; doi:10.3390/ijms242115534)

Figure S1. Average daily feed intake statistics. LW and MS represent Large White pigs and Mashen pigs, respectively; 25 and 4 indicate 25°C and 4°C, respectively.

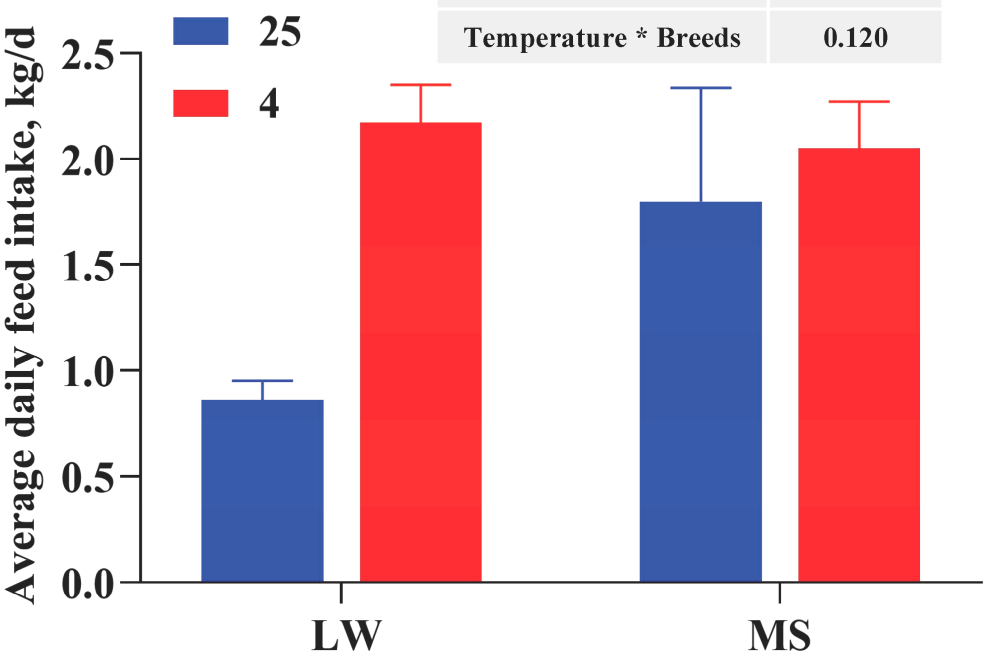

Effect	P-value
Temperature	0.034
Breeds	0.217
Temperature * Breeds	0.120

Figure S2. All DEGs in the transcriptome sequencing data in LW and MS pigs at 25℃ and 4℃. (**A**) Cluster map of DEGs between LW-25 and MS-25 groups. (**B**) Cluster map of DEGs between LW-4 and MS-4 groups. (**C**) Cluster map of DEGs between LW-25 and LW-4 groups. (**D**) Cluster map of DEGs between MS-25 and MS-4 groups. (**E**) Volcanic map of DEGs between LW-25 and LW-4 groups. (**F**) Venn diagram of 4 groups. (G) The number of up-regulated and down-regulated between LW-25 and LW-4 groups. (**H**) The number of up-regulated and down-regulated between MS-25 and MS-4 groups. (I) GO enrichment analysis of specific DEGs between LW-25 and LW-4 groups. (**J**) KEGG pathway classification of specific DEGs between LW-25 and LW-4 groups. LW and MS represent Large White pigs and Mashen pigs, respectively; 25 and 4 indicate 25°C and 4°C, respectively.

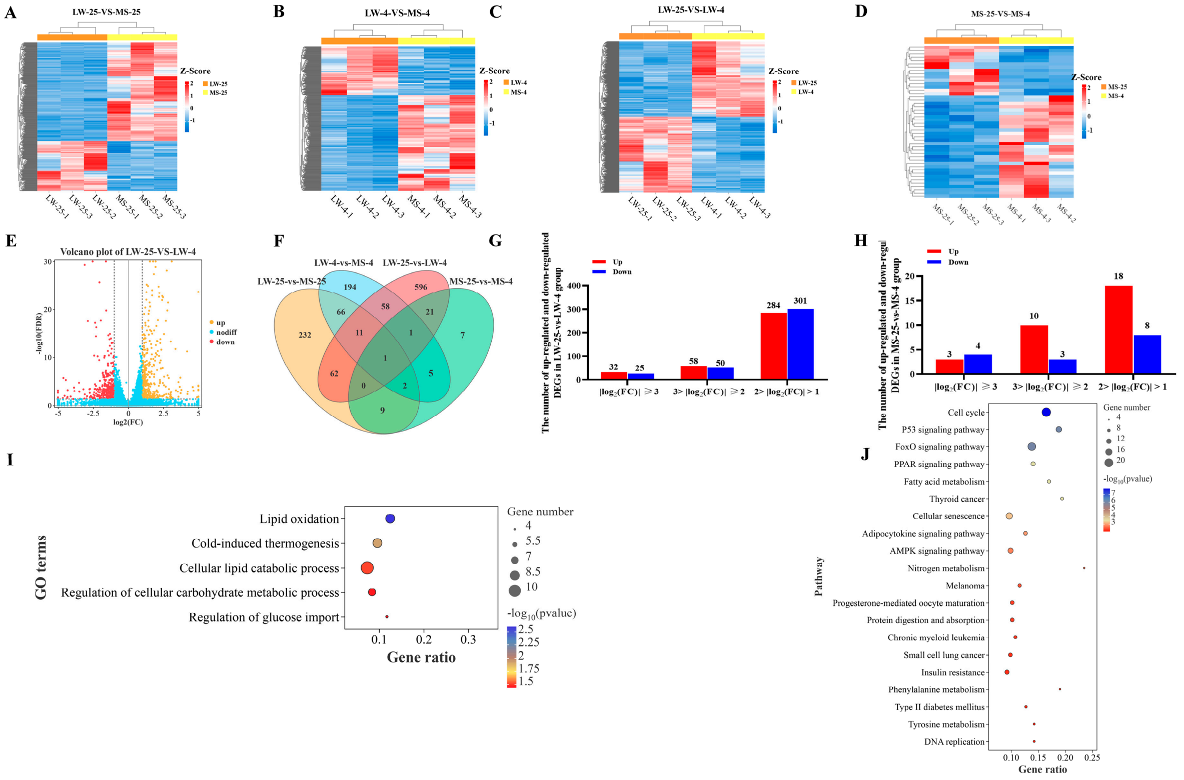

Figure S3. Weighted co-expression network analysis. (**A**) The results of soft threshold β calculation indicated a scale-free network linear model, with network connectivity corresponding to different soft thresholds. The blue line represents a correlation coefficient of 0.8, and the red line represents a correlation coefficient of 0.9. (**B**) Clustering of module eigengenes.

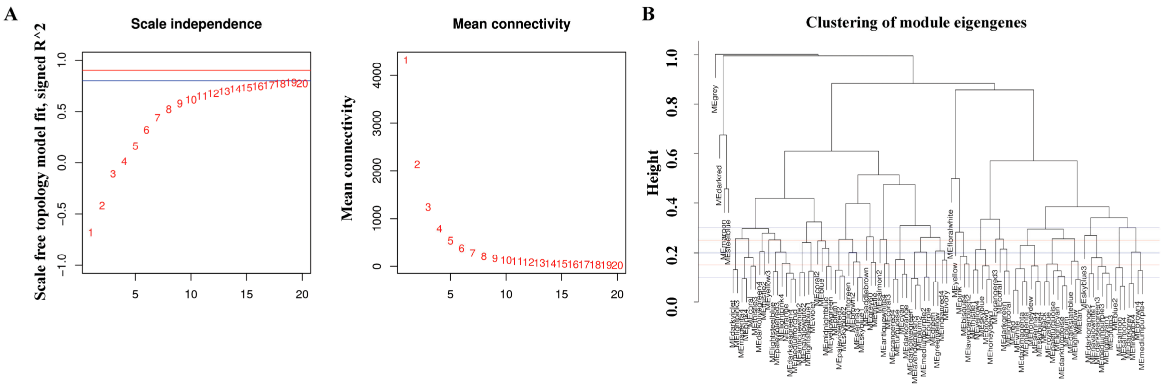

Figure S4. (A–K) qRT-PCR verification on thermogenesis and energy metabolism of longissimus dorsi in LW and MS pigs at 25℃ and 4℃. LW and MS represent Large White pigs and Mashen pigs, respectively. ** $p < 0.01$ and * $p < 0.05$.

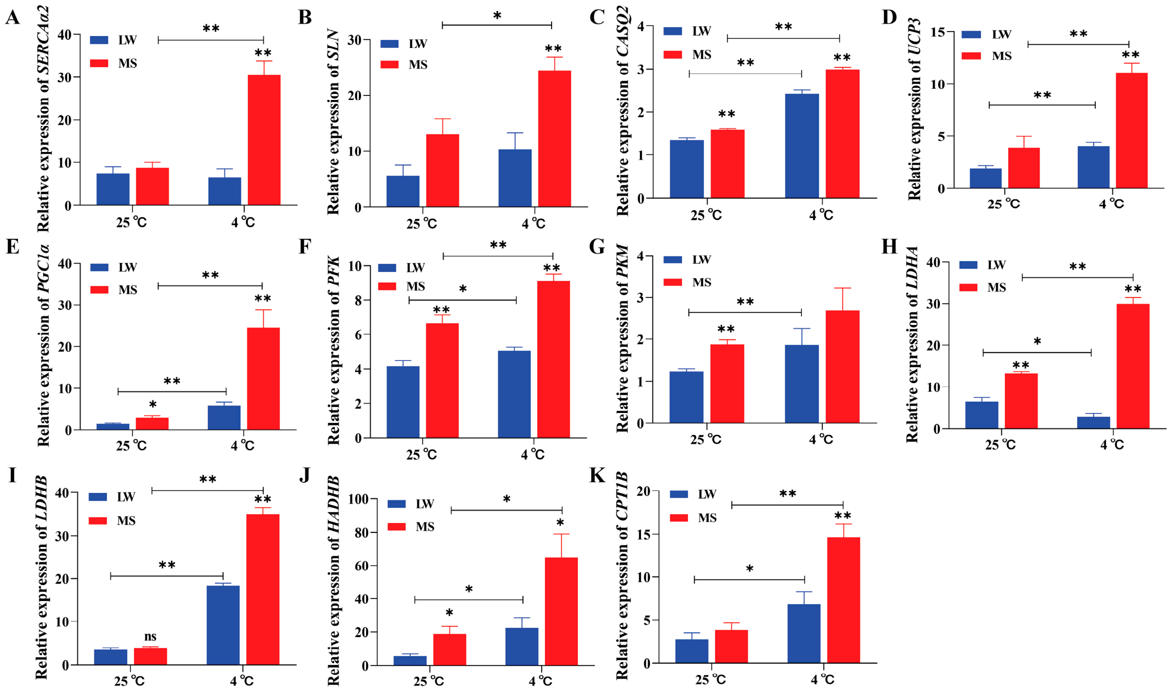

Figure S5. Protein statistical results. (**A**) Western blot statistical results of candidate genes in longissimus dorsi muscle tissues of LW and MS pigs at 25℃ and 4℃. (**B**) Statistical results of heat production and energy metabolism related proteins in porcine skeletal muscle satellite cells interfered with PRSS8. LW and MS represent Large White pigs and Mashen pigs, respectively. ** $p < 0.01$ and * $p < 0.05$.

A

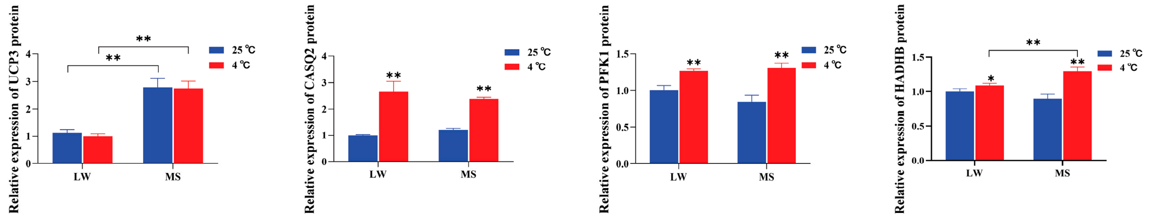

B

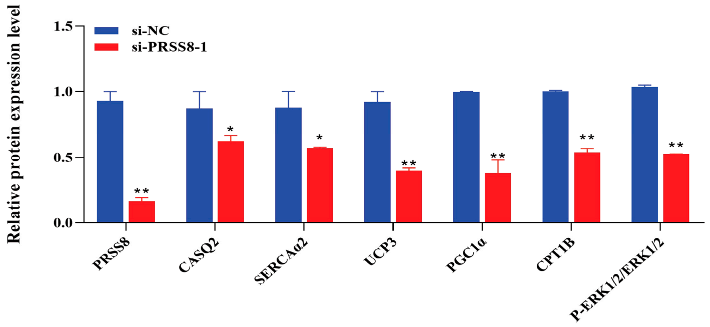

Supplement: Supplementary file 1 [file ijms-24-15534-s001.zip › Supplementary Figures.pdf]
